# Atmospheric observations suggest methane emissions in north-eastern China growing with natural gas use

**DOI:** 10.1038/s41598-022-19462-4

**Published:** 2022-11-17

**Authors:** Fenjuan Wang, Shamil Maksyutov, Rajesh Janardanan, Aki Tsuruta, Akihiko Ito, Isamu Morino, Yukio Yoshida, Yasunori Tohjima, Johannes W. Kaiser, Xin Lan, Yong Zhang, Ivan Mammarella, Jost V. Lavric, Tsuneo Matsunaga

**Affiliations:** 1 National Institute for Environmental Studies, Tsukuba, Japan; 2 Finnish Meteorological Institute, Helsinki, Finland; 3 Deutscher Wetterdienst, Offenbach, Germany; 4 Cooperative Institute for Research in Environmental Sciences, University of Colorado Boulder, Boulder, CO USA; 5 Global Monitoring Laboratory, National Oceanic and Atmospheric Administration, Boulder, USA; 6 Meteorological Observation Center, China Meteorological Administration, Beijing, China; 7 University of Helsinki, Helsinki, Finland; 8 Max Planck Institute for Biogeochemistry, Jena, Germany; 9 Present Address: Acoem Australasia, Melbourne, Australia

**Keywords:** Climate sciences, Environmental sciences

## Abstract

The dramatic increase of natural gas use in China, as a substitute for coal, helps to reduce CO_2_ emissions and air pollution, but the climate mitigation benefit can be offset by methane leakage into the atmosphere. We estimate methane emissions from 2010 to 2018 in four regions of China using the GOSAT satellite data and in-situ observations with a high-resolution (0.1° × 0.1°) inverse model and analyze interannual changes of emissions by source sectors. We find that estimated methane emission over the north-eastern China region contributes the largest part (0.77 Tg CH_4_ yr^−1^) of the methane emission growth rate of China (0.87 Tg CH_4_ yr^−1^) and is largely attributable to the growth in natural gas use. The results provide evidence of a detectable impact on atmospheric methane observations by the increasing natural gas use in China and call for methane emission reductions throughout the gas supply chain and promotion of low emission end-use facilities.

## Introduction

Over the last decade, natural gas (NG) has become the fastest-growing fossil energy in China as a result of coal-to-gas switch action to reduce air pollution and carbon dioxide (CO_2_) emissions. The NG consumption increased dramatically from 108.5 billion standard cubic meters (bcm) in 2010 (4% of primary energy consumption) to a record level of 306.4 bcm in 2019 (8.1% of primary energy consumption), and it will keep increasing according to China’s energy plan, and the share of gas in the energy mix is expected to reach 15% by 2030, while coal and oil consumption will decline^1^. Domestic production of natural gas has increased approximately twofold from 94.8 to 176.2 bcm, and the imported NG also increased dramatically. Methane (CH_4_) is the primary component of NG and the second most important anthropogenic greenhouse gas after CO_2_ with an estimated 20-year global warming potential 84–86 times greater than CO_2_^2^. Oil and natural gas production is one of the major sources of CH_4_ in the atmosphere^3,4^. The CH_4_ leakage rate from NG upstream (extraction and gathering, processing, transmission and storage, distribution) and end-use combustion to the atmosphere is the key factor determining climatic advantage of the coal-to-gas shift^5,6^. Atmospheric measurements studies found that a large amount of methane emissions from oil and gas production are unaccounted for the bottom-up inventories^7,8^. Chan et al.^9^ reported eight-year estimates of methane emissions from oil and gas operations in western Canada and found that they are nearly twice of those from inventories. Zhang et al.^10^ estimated a leakage equivalent to 3.7% (~ 60% higher than the national average leakage rate) of the gross gas extracted from the largest oil-producing basin in the United States (US) using high-resolution satellite observations. Moreover, basin-wide estimates of emissions using in situ airborne data reported an inverse relationship between the basin-level leakage rate and gas production^11^.

Emissions from NG distribution network were found to be the major CH_4_ contributor (56%) accounting for the detectable CH_4_ emissions in Paris, as evidenced from CH_4_ and its isotopic composition by mobile measurement on the ground^12^. Leaks from the NG pipelines were identified as the main source of CH_4_ in emissions in London^13^ and several US cities and the leak rates vary in a large range (from 0.004 leaks/km to 0.63 leaks/km)^14–16^. Advanced mobile leak detection (AMLD) platform combined with GIS information of utility pipeline is used to estimate CH_4_ leakage from pipelines from the local distribution systems in the United States. It is found that the leakage from those pipelines is approximately 5 times greater than inventories based on self-reported utility leakage data^17^. It was also found that the chances of leakage increase with the aging of the pipeline infrastructure irrespective of the material types.

From 2010 to 2019, the length of the gas supply pipelines in the urban areas of China has increased approximately threefold from 298.6 to 935.6 million meters including 82% in the city and 18% in the county seat^18^. The CH_4_ leakage from those pipelines is not actively monitored, which might be a potential threat to the net carbon reduction of China’s energy switch strategy to reach the carbon–neutral goal in 2060^19^. China is the biggest methane emitting country^3^ and many studies report increases in methane emissions from China in the past decade considering China as one region (eg. Zhang et al.^20^, Jackson et al.^21^, Sheng et al.^22^). But there is limited data publicly available on upstream emissions and local distribution of natural gas emissions in China among different subregions. To overcome the limited access to the proprietary data, the combination of surface observations and satellite observations of column-averaged dry-mol fractions of methane provide an opportunity to discover the leaks and to estimate emissions through top-down approaches. Here we use nine years of observations by the GOSAT satellite and the WDCGG (World Data Centre for Greenhouse Gases) surface stations, for the first time to estimate methane emissions in subregions of China from 2010 to 2018 using a high-resolution inverse model and find an impact of increasing natural gas use in China on CH_4_ emissions, signaling a need for effective mitigation strategies.

## Results and discussions

### Regional inversion of CH_4_ emission

**Figure 1:**
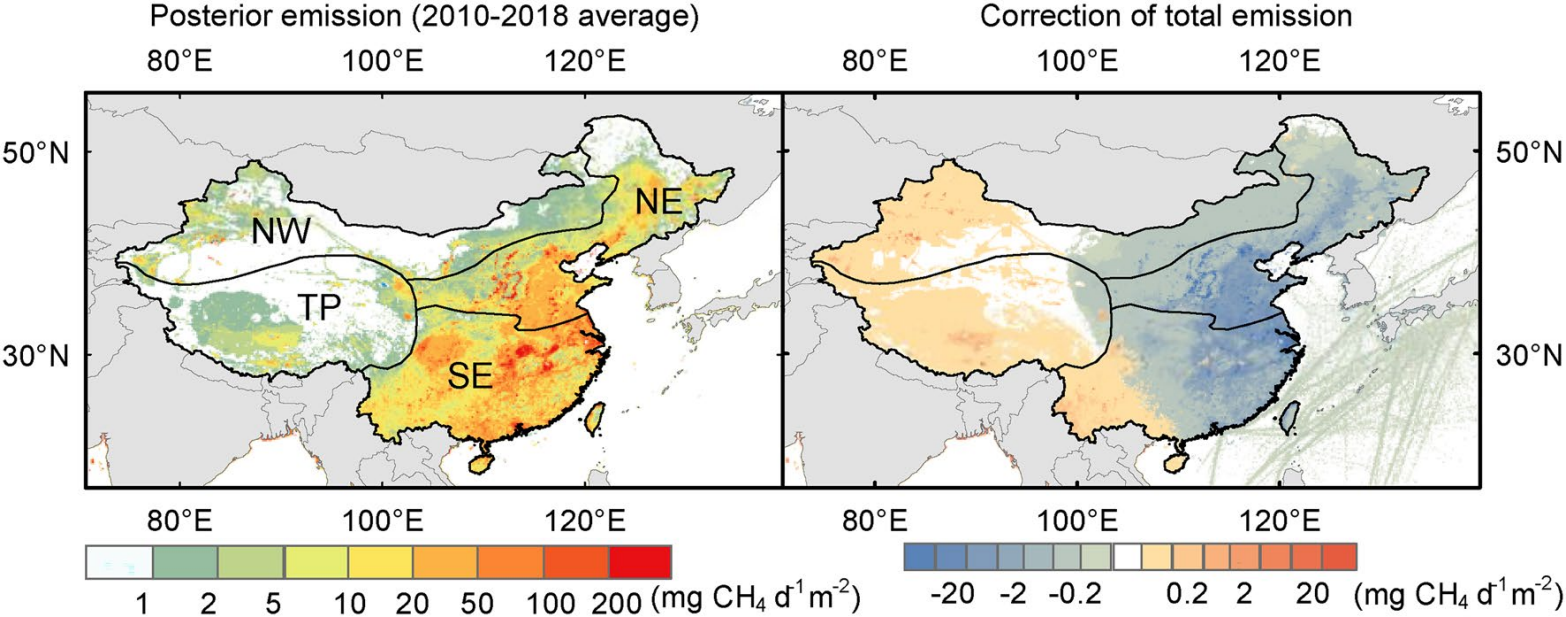
The emission estimates for 2010–2018 and the model corrections to net emissions in China. The four regions are the north-eastern China (NE), the south-eastern China (SE), the north-western China (NW), and the Qinghai-Tibetan Plateau (TP) areas. (Figures generated by ArcGIS Desktop 10.5.1, https://desktop.arcgis.com/en/arcmap/10.5/).

The NIES-TM-FLEXPART-VAR (NTFVAR) global inverse model^23,24^ is used to estimate the CH_4_ emissions constrained by GOSAT and surface observations from 2010 to 2018. Here we focus on the analysis of inverse model results over China and its subregions. There are several ways of regional division of China. In this study, we use a four-region division, based on different geographical features, that is, the north-eastern China (NE), the south-eastern China (SE), the north-western China (NW), and the Qinghai-Tibetan Plateau (TP) areas (Fig. 1). These regions differ in climate, agriculture type, also differ in the major economic activities and CH_4_ emission sources. The NE and SE regions are in the Eastern monsoon area and are divided by the Qingling Mountains-Huai River, which is also the dividing line of 800 mm mean annual precipitation, with the SE region experiencing more precipitation. Daxinganling-Yinshan-Helan mountain is the physical geographic boundary of North and Northwest, which is also the dividing line of 400 mm mean annual precipitation. The Northwest region is a non-monsoon area with mean annual precipitation of less than 400 mm, including Xinjiang and Inner Mongolia where the main agriculture is animal husbandry^25^. TP is a region at an average elevation over 4000 m, with the Kunlunshan range, Qilianshan range, and Hengduan mountain chain as the division to other three regions.

Our optimized estimate of the average Chinese emissions is 58 Tg CH_4_ yr^−1^ during 2010–2018 (with model uncertainty of 8.6 Tg CH_4_ yr^−1^), around 12% lower than the average prior emission of 65.6 Tg CH_4_ yr^−1^, and our estimate is consistent with Sheng’s^22^ top-down study with 57.6 Tg CH_4_ yr^−1^ over 2010–2017. The optimized average CH_4_ budgets from the four subregions over 2010–2018 are 30.0 $\pm$ 1.0 (average $\pm$ standard deviation) Tg CH_4_ yr^−1^ from SE, 23.3 $\pm$ 2.7 Tg CH_4_ yr^−1^ from NE, 2.9 $\pm$ 0.2 Tg CH_4_ yr^−1^ from NW, and 1.7 $\pm$ 0.1 Tg CH_4_ yr^−1^ from TP. NE and SE emit an order of magnitude more CH_4_ compared to NW and TP. The inverse model corrections are opposite in western and eastern China as illustrated in Fig. 1. Prior emissions in TP are underestimated, especially in the west part of TP by 15–30%. Emissions are also underestimated in the west part of NW where main flux hotspots depict the oil production sites. Prior emissions in the eastern coastal regions, however, are overestimated by 15–25%, where high density and a large quantity of fluxes are shown. The scale of the adjustment in different regions are within the uncertainty range estimated for other countries^26^. The overestimation of anthropogenic emissions from China has been reported by previous inversion studies using former versions of EDGAR inventories^20,27–29^. Our high-resolution inversion suggests that the overestimation originates from the western part of China while its eastern part is underestimated. The improvement of model fit to the observations by the inverse model is confirmed by an independent model evaluation with in-situ measurement and airplane observations (shown in Supplementary Table 1).

### Interannual changes in CH_4_ emissions

**Figure 2:**
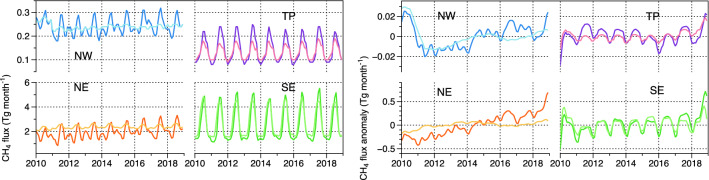
CH_4_ flux interannual variability and 2010–2018 anomaly in four regions NE, SE, NW, and TP in China, lighter colour standing for prior emission and darker colour standing for posterior emission in each region.

Figure 2 shows the seasonal variation and anomalies of prior and posterior total emissions in the four regions. The seasonal variability in TP and SE with maximum emissions in summer and minimum in winter corresponds to wetlands and rice paddies, since SE is the main rice production area of China^30^. A pronounced peak in summer and the second peak in winter is evident both in NW and NE, where residential heat is supplied in winter. The posterior fluxes in NW, TP, and NE show large variability compared to the prior fluxes.

The anomalies in interannual variability are calculated by subtracting the long-term monthly mean flux from the raw time series and centred running mean (11-month) is constructed on the resultant time series to smooth out short-term (monthly) fluctuations and highlight longer-term (yearly) trends. The posterior flux of TP shows a notable positive anomaly in 2011 in response to the La Nina event during 2010–2011 and a negative anomaly in response to the strong El Nino event during 2015–2016. A decreasing trend is detected during the transition from 2011 La Nina to 2016 El Nino in the posterior flux in TP where the natural emissions are dominated, and similar trend was reported in Southern Asia during the period^31^. An observation study also found that the atmospheric CH_4_ concentration in TP increased rapidly during 2010–2012 and slowly during 2013–2015^32^. The rapid increases in the latter half of 2018 in all regions may be due to using 11-month running mean instead of signal from the inversion and the variation in NW need to be further investigated.

Statistically significant increase trends in 2010–2018 are detected in NE for both prior and posterior flux anomalies of total CH_4_ emissions (95% confidence P ≤ 0.05 by Mann–Kendall approach^33^). The increasing trend in SE is weaker but still significant (95% confidence). No trends in TP and NW are found. We estimate a yearly increase rate of 0.87 Tg CH_4_ yr^−1^ for the whole of China during 2010–2018, which is consistent with Zhang’s^20^ simulation of 0.72 Tg CH_4_ yr^−1^ for China during 2010–2016, but lower than the estimate of Miller’s^28^ with 1.1 Tg CH_4_ yr^−1^ over 2010–2015. NE contributes the most to the growth rate (0.77 Tg CH_4_ yr^−1^) followed by SE (0.13 Tg CH_4_ yr^−1^), which is stronger compared to the increase of the prior fluxes (0.35 Tg CH_4_ yr^−1^).

### Trends in regional emission sectors

**Figure 3:**
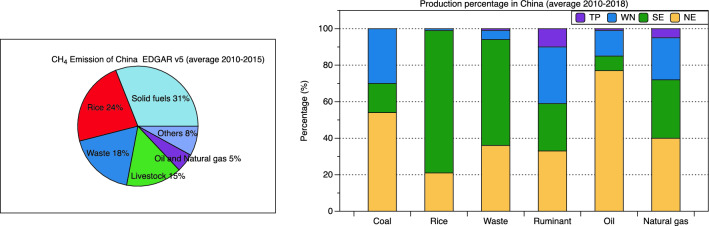
Prior anthropogenic CH_4_ emission from sectors and related production percentage in four regions.

Anthropogenic CH_4_ emission is about 90% of the total CH_4_ emission in China^31,34^. Figure 3 shows the relative contributions of major CH_4_ emission sources in China and related productions percentage in the four regions analysed in this study using data from EDGAR v5 and China National Statistic Yearbook^18^. The major anthropogenic emission sources are solid fuel (31%), rice production (24%), waste (18%), and livestock (15%). NE is the most energy production region producing 55, 77, and 40% of Chinese national coal, oil, and natural gas each year, followed by NW which produces 29, 14, and 23% of Chinese national coal, oil and natural gas each year. 77% of rice is produced in SE and 21% in NE. The total volume of collected municipal solid waste and wastewater discharged are mainly in SE (60%) and NE (35%) due to its large population in SE (58% of national population) and NE (36% of national population). Ruminant population spreads in the four regions with 33% in NE, 31% in NW, 26% in SE, and 10% in TP.

Coal production in China peaked in 2012 and declined since then from 41 to 37 Gt yr^−1^ until 2018 with a significant decrease in SE (decrease from 7.5 to 4 Gt yr^−1^).^18^ Rice production, ruminant population, and crude oil production remain relatively stable (Supplementary Fig. S1). Waste (volume of discharged sewage and domestic removed solid waste) and natural gas production show a dramatic increase during 2010–2018, which might be the major contributors to CH_4_ emission changes. The increase of CH_4_ emissions from waste in China is 0.40 $\pm$ 0.08 Tg CH_4_ yr^−1^ during 2010–2018 (according to EDGAR v6.0^3^), with 60% occurring in SE counterweighing the decrease in emissions caused by coal production. The increase in total CH_4_ emissions in SE is not as significant as it is in NE since CH_4_ emission in SE from coal production decrease significantly and counterweighs the emissions from increasing waste and NG. The estimated CH_4_ emission from coal production in SE is 0.36 Tg CH_4_ yr^−1^ with a national average emission factor of 9.3 m^3^ t^−1^, while previous studies suggested that the coal mines with high methane content have emission factors over 20 m^3^ t^−1^ in SE, which indicates that both the coal emissions and their decline can be even larger^35,36^.

### Contribution of natural gas emission in north-eastern China

**Figure 4:**
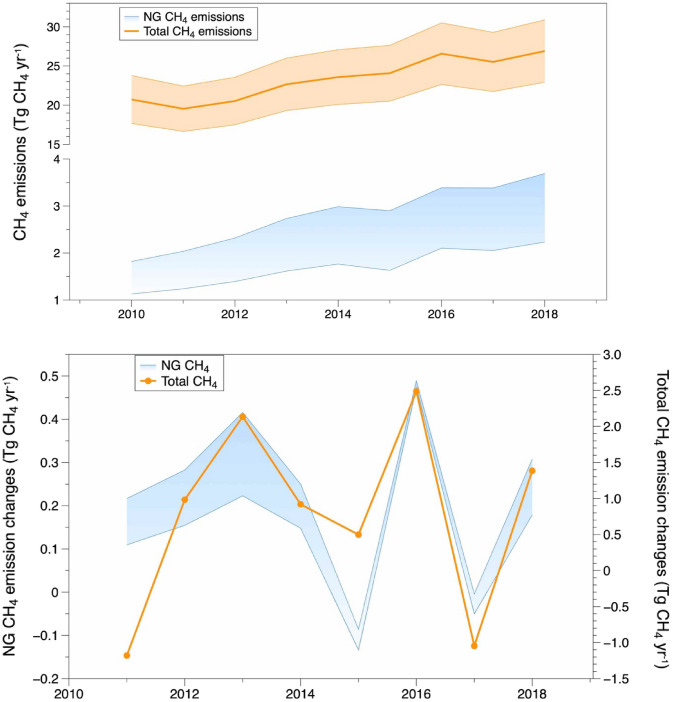
Estimated CH_4_ emissions from NG (up and low range) and total CH_4_ emission (estimation in a solid line with uncertainty in shadow in NE during 2010–2018 (left panel) (detailed data in Supplementary Table S2) and CH_4_ emission increment relative to previous year (right panel).

Estimation of CH_4_ emissions from NG includes leakage from energy extraction, processing, transport, and leakage at end-use applicant according to site measurements and province-level data on pipeline distribution leakage provided by NG suppliers (see Methods). The left panel in Fig. 4 shows the upper and lower bounds of the estimates of CH_4_ emission from NG and the total CH_4_ emission trend with proportioned uncertainty during 2010–2018 in NE, both of which depict statistically significant increasing trends. The variation of total CH_4_ emission increase closely follows the changes of CH_4_ emissions from NG (Fig. 4 right panel), indicating NG leakage of CH_4_ emissions is a notable driver of CH_4_ emission increase in NE. Removing the waste sector increment (0.14 Tg CH_4_ yr^−1^) estimated by EDGAR v6.0 from the total increase in NE, we estimate an average CH_4_ emission growth of 0.63 Tg CH_4_ yr^−1^ in NE. The estimated NG leakage contributes 0.14 ~ 0.23 Tg CH_4_ yr^−1^, and the changes of pipeline leakage dominate the variation of CH_4_ emissions from NG which is accounted from province-level loss amount from gas supply pipeline. Natural gas pipeline leaks (estimated by the difference between the amount of gas purchased and the amount of gas sold) is 3.4% in NE and 2.7% in China during 2010–2018, which is higher than the estimate of ~ 1.4% in Russia^37^ and within the range of British estimate between 1.9 and 10.8%^38^. Taking 2018 year as example, in NE region the NG production is 63 bcm, and the NG consumption is 101.5 bcm^18^. The estimated total NG emission is 5.2 ~ 8.6% of the regional NG production or 3.2 ~ 5.3% of the regional NG consumption. A previous study^39^ has found that the self-accounting by NG supplies potentially can be lower than the actual leakage. The discrepancy between top-down and bottom-up estimations of CH_4_ emissions implies that a significant amount of CH_4_ leaks are not accounted for. Our estimates of the growing trend are higher than it in the inventory, and some other studies suggest that CH_4_ emissions from NG use can be underestimated by the bottom-up approach in China^40^. The analysis shows a strong correlation between the trend in NG use and the increase of the CH_4_ concentration over NE China, translated into emission changes by the inverse model.

### Implications for natural gas emission mitigation

Our analysis shows that the top-down approach based on inversion modeling can support an observation-driven assessment of methane emissions from methane leakage, especially GOSAT data providing a long-term trend. Results highlight the relevance of NG use and pipeline expansion to methane emissions. Such an increase of leaking methane from NG production and use chain will cause potential danger to diverse stakeholders despite introducing a net carbon reduction.

Given the large NG distribution pipelines 935.6 million meters in China, NG leakage can be a significant waste of energy and money. It can also accelerates ozone formation in urban areas^41^, especially in North China where surface ozone is already a severe air pollution problem^42,43^. Increase the monitoring of NG CH_4_ emissions through a number of methods from facility-level measurements^44^ to city-scale surveys^39,45,46^ is a pressing task to successful mitigation strategy. Advanced leak reduction technologies in the NG end-use sector can also bring economic, environmental, and health benefits^17,46^.

## Methods

### Methane observations

Atmospheric CH_4_ observations from satellite, surface, aircraft, and ship platforms are used in this study. Greenhouse Gases Observing Satellite (GOSAT) is the first satellite dedicated to observing greenhouse gases from space launched in January 2009^47^. The orbit overpasses at around 12:49 (local time) every three days, and the diameter of the footprint in nadir is approximately 10.5 km. The Thermal and Near-infrared Sensor for carbon Observation-Fourier Transform Spectrometer (TANSO-FTS) is the main instrument of GOSAT, measuring short-wavelength infrared (SWIR) radiance reflected from the Earth’s surface and atmosphere. We use column-averaged dry-air mole fraction of methane (XCH_4_) data from the NIES GOSAT Level 2 retrievals (v. 02.81)^48^. The GOSAT XCH_4_ data is further corrected by subtracting the monthly mean difference for each 5° latitude band between GOSAT observations and the model simulated XCH_4_ optimized with inversion that uses only surface observations^31^. Ground-based atmospheric CH_4_ observation data are obtained from WDCGG, and aircraft and ships observations are from NIES (map shown in Supplementary Fig. S2). Weekly flask-air samples and continuous measurements are contained in the ground-based atmospheric CH_4_ observation data. The data from the flask sampling sites are used as an average concentration for a pair of flasks. For the discrete flask-air measurements with pair sampling, average mole fraction for the pair is used. The continuous observations are first averaged at hourly scale. Hourly data within 12:00–16:00 LT with well-mixed conditions (except for mountain sites, where 0:00–4:00 LT) is used to represent daily averages as model inputs. Rejection thresholds are set for data of GOSAT and the ground-based sites to filter out outliers (detailed description at Wang et al.^23^).

### Atmospheric inverse model

We use the joint Eulerian three-dimensional transport model coupled with a Lagrangian model FLEXPART (FLEXible PARTicle dispersion model) as the Lagrangian Particle Dispersion Model (LPDM)^49,50^. The coupled model NIES-TM-FLEXPART-VAR (NTFVAR) combines National Institute for Environmental Studies Transport Model (NIES-TM) v08.1i with a horizontal resolution of 2.5° and 32 hybrid-isentropic vertical levels^51^ and FLEXPART model v.8.0^52^. The transport model is driven by the meteorological data from the Japanese Meteorological Agency (JMA) Climate Data Assimilation System (JCDAS)^53,54^. In this study, variational inversion scheme is combined with the high-resolution variant of the transport model and its adjoint described by Maksyutov et al.^24^, and applied to inverse modelling of methane emissions in a number of studies^4,21,31^. The inverse modeling problem is formulated and solved to find the optimal value of corrections to prior fluxes considering mismatches of observations and modelled concentrations. Variational optimization is applied to obtain flux corrections as anthropogenic and wetland scaling factors to vary prior uncertainty fields on a monthly basis at a 0.1° × 0.1° resolution separately for anthropogenic and natural wetland emissions with bi-weekly time steps. The inverse model operates at the resolution of coupled transport model of 0.1° × 0.1° and applies spatial flux covariance length of 500 km. Uncertainty tests for the inverse model have been performed using randomly perturbed observations and perturbed fluxes for different regions. We perturbed five sets of observations consistently with the observation uncertainty at each site and produced five sets of perturbed monthly Emissions Database for Global Atmospheric Research (EDGAR) and VISIT fluxes with a random scaling factor applied separately for regions and each month. We then performed an inversion using the perturbed pseudo-observations as measurement data and the perturbed fluxes (perturbed EDGAR and VISIT combined with the non-perturbed soil sink, biomass burning, and other natural emissions from the ocean, geological sources, and termites) as the prior fluxes and compared the inversion results to get the standard deviation of the estimated emissions. The uncertainty of inverse simulation in China is 16.5%^23,24^. Independent evaluation of the inverse model was made by observations at Dongsha Island (DSI) (20.7 N, 116.7E) and Novosibirsk (NOV) (55 N, 83E). DSI is located to the south of China and NOV is to the north of China (shown in Supplementary Fig. S1). Bias and Root Mean Squared Error (RMSE) between observed and modelled concentrations with posterior fluxes decrease in both sites, compared to the modelled concentrations with prior fluxes (Supplementary S2).

A combination of emission inventories is used as prior fluxes. Annual anthropogenic emissions are from the EDGAR v5. EDGAR provides a grid map at 0.1° × 0.1° resolution at the global level, and emissions of CH_4_ include all sources, such as fossil fuel production, agriculture, wastes and so on^55,56^. The monthly variation of anthropogenic emissions is based on the EDGAR climatology data of 2015. EDGAR v5 updates emissions until 2015. Data beyond 2015 are extended by proportional scaling of EDGAR values of 2015 to the respective yearly values in the report of PBL Netherlands Environmental Assessment Agency^57^. Wetlands emissions are from VISIT model simulations^58^. Biomass burning emissions are from the daily Global Fire Assimilation System (GFASv1.2)^59^. Climatological emissions from the oceanic^60^, geological^61^, and termite^62^ sources are also included.

### Estimation of natural gas emissions

As a comparison, fugitive CH_4_ emissions from NG systems are estimated on the basis of the 2006 IPCC Guidelines for Greenhouse Gas Inventories, and recent measurements. The province-level annual natural gas production data is collected from the China Statistical Yearbook^18^ (2010–2018) (see Supplementary Fig. S3 for more details). The fugitive emissions rates (FERs) for NG systems upstream (energy extraction, processing, transport, and distribution) in China is set as constant at 1.8% (0.35kt CH_4_ PJ^−1^) for the 2010–2018 period taken from Schwietzke’s^63^ study. There exist only limited measurements of gas leakage in China. The conventional gas methane leakage rates reported in the latest U.S. field studies are applied to China. Alvarez et al.^8^ found agreement between site-level results and top-down results, with the best estimate of supply chain emissions. This estimate of oil/NG CH_4_ emissions can also be expressed as a production-normalized emission rate of 2.3% (+ 0.4%/− 0.3%) by normalizing annual gross natural gas production, which is mainly from production, gathering, and processing sources. Zhang et al.^10^ estimated natural gas production emission rate (or methane leakage rate) of 3.7 $\pm$ 0.7% by a high-resolution satellite data-based atmospheric inversion framework which is ~ 60% higher than the national average of 2.3 $\pm$ 0.3% in the largest oil and gas production basin in the US. The leakage rate is even higher for the rapidly developing Delaware sub-basin (4.1%). The emission distribution is based on province-level loss amount from gas supply pipeline, defined as the difference between the amount of gas purchased (e.g., what enters the gateway to a province) and the amount of gas sold (e.g., what is metered to consumers), obtained from the China City Statistical Yearbook (2010–2018)^64^ (see Supplementary Fig. S3 for more details). End-use (power generation, residential cooking, and industrial boilers) processes included in the estimation is 0.4–0.9% following by Lebel’s^65^ estimation of appliance level leakage. Taking the upstream and end-use EFs into account, the CH_4_ emission is estimated as the sum of low FERs (1.8%) and high FERs (4.4%) for production, 0.4–0.9% of end-user combustion, and the accounted province-level loss.

## Supplementary Information

Supplementary Information.

## Data Availability

The inverse model and forward transport model code can be made available to potential research collaborators upon reasonable request. The XCH_4_ retrievals (NIES Level 2 retrievals) are available at: https://data2.gosat.nies.go.jp. Furthermore, in-situ data are archived on the WDCGG Global Network: https://gaw.kishou.go.jp (more details on data file information and references see Supplementary S4). Other data products used in the study like the EDGAR emissions inventory and GFAS Database are available for download at http://edgar.jrc.ec.europa.eu/ and https://www.ecmwf.int/en/forecasts/dataset/global-fire-assimilation-system. Wetland emission VISIT are available at https://www.nies.go.jp/doi/10.17595/20210521.001-e.html, and the NIES airborne and JR STATION (Japan-Russia Siberian Tall Tower Inland Observation network) data are available at https://db.cger.nies.go.jp/portal/geds/atmosphericAndOceanicMonitoring. Ship data are available by request to NIES observation group.

## References

[1] Energy Production and Consumption Revolution Strategy (2016–2030). https://www.ndrc.gov.cn/fggz/zcssfz/zcgh/201704/W020190910670685518802.pdf (China national development and reform commission, 2016).

[2] IPCC & Stocker, T. F. Q. *et al. Climate Change 2013: The Physical Science Basis. Contribution of Working Group I to the Fifth Assessment Report of the Intergovernmental Panel on Climate Change* (2013).

[3] Crippa M (2020). High resolution temporal profiles in the emissions database for global atmospheric research. Sci. Data.

[4] Saunois M (2020). The global methane budget 2000–2017. Earth Syst. Sci. Data.

[5] Tanaka K, Cavalett O, Collins W, Cherubini F (2019). Asserting the climate benefits of the coal-to-gas shift across temporal and spatial scales. Nat. Clim. Change.

[6] Qin Y, Edwards R, Tong F, Mauzerall D (2017). Can switching from coal to shale gas bring net carbon reductions to China?. Environ. Sci. Technol..

[7] Brandt A (2014). Methane leaks from north American natural gas systems. Science.

[8] Alvarez R (2018). Assessment of methane emissions from the US oil and gas supply chain. Science.

[9] Chan E (2020). Eight-year estimates of methane emissions from oil and gas operations in western Canada are nearly twice those reported in inventories. Environ. Sci. Technol..

[10] Zhang Y (2020). Quantifying methane emissions from the largest oil-producing basin in the United States from space. Sci. Adv..

[11] Peischl J (2018). Quantifying methane and ethane emissions to the atmosphere from central and western US oil and natural gas production regions. J. Gerontol. Ser. A Biol. Med. Sci..

[12] Defratyka S (2021). Mapping urban methane sources in Paris, France. Environ. Sci. Technol..

[13] Zazzeri G (2017). Evaluating methane inventories by isotopic analysis in the London region. Sci. Rep..

[14] McKain K (2015). Methane emissions from natural gas infrastructure and use in the urban region of Boston, Massachusetts. Proc. Natl. Acad. Sci. U. S. A..

[15] Lamb B (2016). Direct and indirect measurements and modeling of methane emissions in Indianapolis, Indiana. Environ. Sci. Technol..

[16] von Fischer J (2017). Rapid, vehicle-based identification of location and magnitude of urban natural gas pipeline leaks. Environ. Sci. Technol..

[17] Weller Z, Hamburg S, von Fischer J (2020). A national estimate of methane leakage from pipeline mains in natural gas local distribution systems. Environ. Sci. Technol..

[18] *China Statistical Yearbook*. (China Statistics Press, 2010–2018).

[19] Mallapaty S (2020). How China could be carbon neutral by mid-century. Nature.

[20] Zhang Y (2021). Attribution of the accelerating increase in atmospheric methane during 2010–2018 by inverse analysis of GOSAT observations. Atmos. Chem. Phys..

[21] Jackson RB (2020). Increasing anthropogenic methane emissions arise equally from agricultural and fossil fuel sources. Environ. Res. Lett..

[22] Sheng J (2021). Sustained methane emissions from China after 2012 despite declining coal production and rice-cultivated area. Environ. Res. Lett..

[23] Wang F (2019). Methane emission estimates by the global high-resolution inverse model using national inventories. Remote Sens..

[24] Maksyutov S (2020). Atmos. Chem. Phys..

[25] Song Y, Achberger C, Linderholm HW (2011). Rain-season trends in precipitation and their effect in different climate regions of China during 1961–2008. Environ. Res. Lett..

[26] Deng Z (2021). Comparing national greenhouse gas budgets reported in UNFCCC inventories against atmospheric inversions. Earth Syst. Sci. Data.

[27] Turner A (2015). Estimating global and North American methane emissions with high spatial resolution using GOSAT satellite data. Atmos. Chem. Phys..

[28] Miller S (2019). China’s coal mine methane regulations have not curbed growing emissions. Nat. Commun..

[29] Maasakkers J (2019). Global distribution of methane emissions, emission trends, and OH concentrations and trends inferred from an inversion of GOSAT satellite data for 2010–2015. Atmos. Chem. Phys..

[30] Zhang G (2020). Fingerprint of rice paddies in spatial–temporal dynamics of atmospheric methane concentration in monsoon Asia. Nat. Commun..

[31] Wang F (2021). Interannual variability on methane emissions in monsoon Asia derived from GOSAT and surface observations. Environ. Res. Lett..

[32] Feng T, Yang Y, Xie S, Dong J, Ding L (2017). Economic drivers of greenhouse gas emissions in China. Renew. Sustain. Energy Rev..

[33] Hirsch, R., Slack, J. & Smith, R. 107–121, 162 (*Water Resources Research* 18.1. ISI Document Delivery No.: NC504, 1982).

[34] Ito A (2019). Methane budget of East Asia, 1990–2015: A bottom-up evaluation. Sci. Total Environ..

[35] Sheng J, Song S, Zhang Y, Prinn R, Janssens-Maenhout G (2019). Bottom-up estimates of coal mine methane emissions in China: A gridded inventory, emission factors, and trends. Environ. Sci. Technol. Lett..

[36] Zhu T, Bian W, Zhang S, Di P, Nie B (2017). An improved approach to estimate methane emissions from coal mining in China. Environ. Sci. Technol..

[37] Leliveld J (2005). Greenhouse gases: Low methane leakage from gas pipelines. Nature.

[38] Mitchell C, Sweet J, Jackson T (1990). A study of leakage from the UK natural-gas distribution-system. Energy Policy.

[39] Jackson R (2014). Natural gas pipeline leaks across Washington, DC. Environ. Sci. Technol..

[40] Gong S, Shi Y (2021). Evaluation of comprehensive monthly-gridded methane emissions from natural and anthropogenic sources in China. Sci. Total Environ..

[41] West J, Fiore A, Horowitz L, Mauzerall D (2006). Global health benefits of mitigating ozone pollution with methane emission controls. Proc. Natl. Acad. Sci. U. S. A..

[42] Wang T (2017). Ozone pollution in China: A review of concentrations, meteorological influences, chemical precursors, and effects. Sci. Total Environ..

[43] Li K (2021). Ozone pollution in the North China Plain spreading into the late-winter haze season. Proc. Natl. Acad. Sci..

[44] Zimmerle D (2015). Methane emissions from the natural gas transmission and storage system in the United States. Environ. Sci. Technol..

[45] Ars S (2020). Investigation of the spatial distribution of methane sources in the greater Toronto area using mobile gas monitoring systems. Environ. Sci. Technol..

[46] Phillips NG (2013). Mapping urban pipeline leaks: Methane leaks across Boston. Environ. Pollut..

[47] Yokota T (2009). Global concentrations of CO2 and CH4 retrieved from GOSAT: First preliminary results. Sola.

[48] Yoshida Y (2013). Improvement of the retrieval algorithm for GOSAT SWIR XCO2 and XCH4 and their validation using TCCON data. Atmos. Meas. Tech..

[49] Ganshin A (2012). A global coupled Eulerian-Lagrangian model and 1 x 1 km CO2 surface flux dataset for high-resolution atmospheric CO2 transport simulations. Geosci. Model Dev..

[50] Belikov D (2016). Adjoint of the global Eulerian-Lagrangian coupled atmospheric transport model (A-GELCA v1.0): Development and validation. Geosci. Model Dev..

[51] Belikov D (2013). Simulations of column-averaged CO2 and CH4 using the NIES TM with a hybrid sigma-isentropic (sigma-theta) vertical coordinate. Atmos. Chem. Phys..

[52] Stohl A, Forster C, Frank A, Seibert P, Wotawa G (2005). Technical note: The Lagrangian particle dispersion model FLEXPART version 6.2. Atmos. Chem. Phys..

[53] Onogi K (2007). The JRA-25 reanalysis. J. Meteorol. Soc. Jpn.

[54] Kobayashi S (2015). The JRA-55 reanalysis: General specifications and basic characteristics. J. Meteorol. Soc. Jpn.

[55] Janssens-Maenhout G (2019). Earth Syst. Sci. Data.

[56] Crippa M (2018). Earth Syst. Sci. Data.

[57] Olivier, J. G. J. & Peters, J. A. H. W. Trends in global CO_2_ and total greenhouse gas emissions: 2018 Report (2018).

[58] Ito A, Inatomi M (2012). Use of a process-based model for assessing the methane budgets of global terrestrial ecosystems and evaluation of uncertainty. Biogeosciences.

[59] Kaiser J (2012). Biomass burning emissions estimated with a global fire assimilation system based on observed fire radiative power. Biogeosciences.

[60] Lambert G, Schmidt S (1993). Reevaluation of the oceanic flux of methane-uncertainties and long-term variations. Chemosphere.

[61] Etiope G, Milkov A (2004). A new estimate of global methane flux from onshore and shallow submarine mud volcanoes to the atmosphere. Environ. Geol..

[62] Fung I (1991). 3-Dimentsional models synthesis of the global methane cycle. J. Gerontol. Ser. A Biol. Med. Sci..

[63] Schwietzke S, Griffin W, Matthews H, Bruhwiler L (2014). Global bottom-up fossil fuel fugitive methane and ethane emissions inventory for atmospheric modeling. Acs Sustain. Chem. Eng..

[64] *China City Statistical Yearbook* (China Statistics Press, 2010–2018).

[65] Lebel E, Lu H, Speizer S, Finnegan C, Jackson R (2020). Quantifying methane emissions from natural gas water heaters. Environ. Sci. Technol..

