# Publisher Correction: Atmospheric observations suggest methane emissions in north-eastern China growing with natural gas use

**DOI:** 10.1038/s41598-023-28548-6

**Published:** 2023-02-02

**Authors:** Fenjuan Wang, Shamil Maksyutov, Rajesh Janardanan, Aki Tsuruta, Akihiko Ito, Isamu Morino, Yukio Yoshida, Yasunori Tohjima, Johannes W. Kaiser, Xin Lan, Yong Zhang, Ivan Mammarella, Jost V. Lavric, Tsuneo Matsunaga

**Affiliations:** 1 National Institute for Environmental Studies, Tsukuba, Japan; 2 Finnish Meteorological Institute, Helsinki, Finland; 3 Deutscher Wetterdienst, Offenbach, Germany; 4 Cooperative Institute for Research in Environmental Sciences, University of Colorado Boulder, Boulder, CO USA; 5 Global Monitoring Laboratory, National Oceanic and Atmospheric Administration, Boulder, USA; 6 Meteorological Observation Center, China Meteorological Administration, Beijing, China; 7 University of Helsinki, Helsinki, Finland; 8 Max Planck Institute for Biogeochemistry, Jena, Germany; 9 Present Address: Acoem Australasia, Melbourne, Australia

Correction to: *Scientific Reports* https://doi.org/10.1038/s41598-022-19462-4, published online 17 November 2022

The original version of this Article contained an error in the upper panel of Figure 4, where the Total CH_4_ emission was inadvertently shown in three lines instead of in a solid line with the uncertainty displayed in orange shadow. The original Figure 4 and accompanying legend appear below.

**Figure 4:**
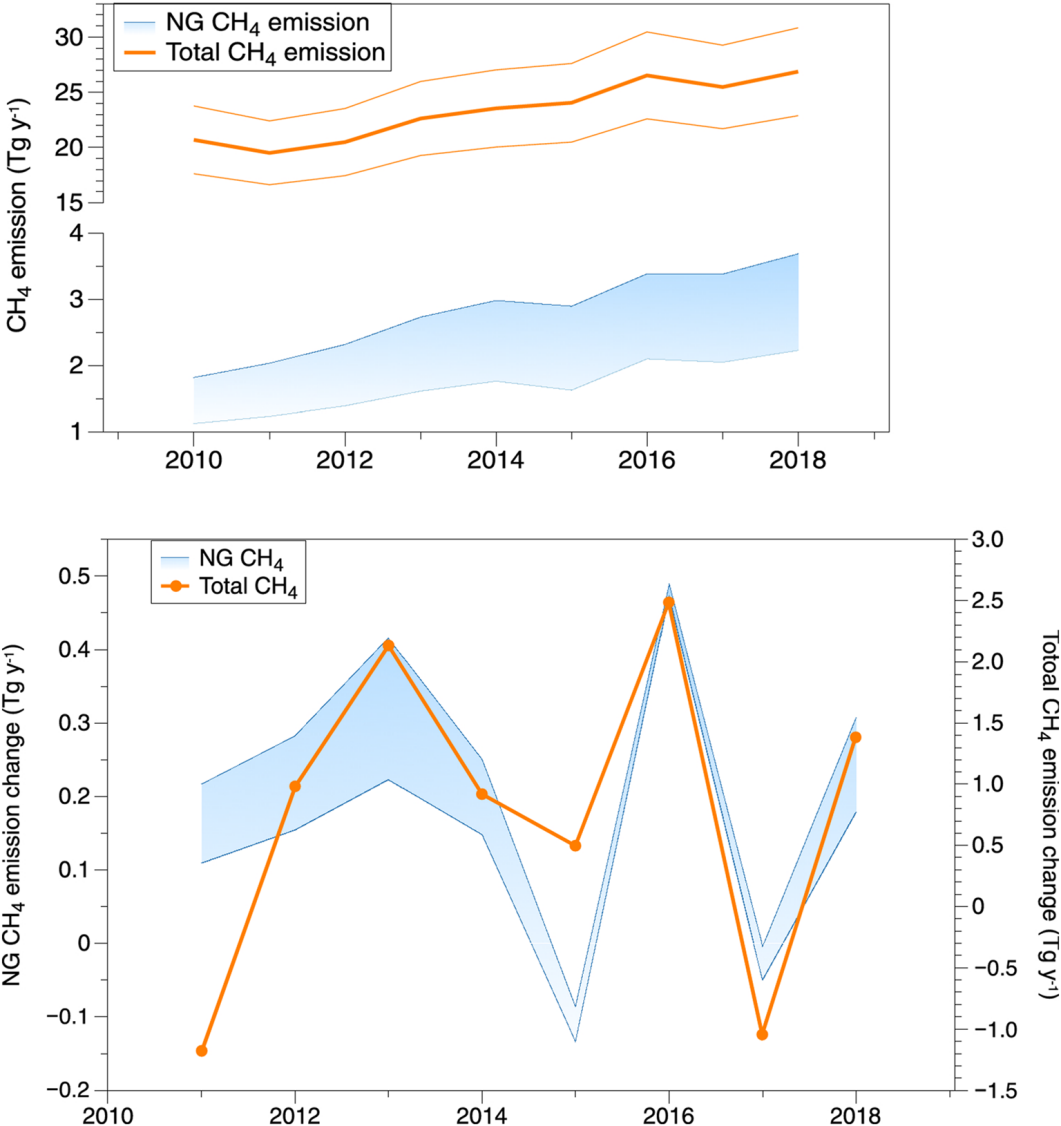
Estimated CH_4_ emissions from NG (up and low range) and total CH_4_ emission (estimation in a solid line with uncertainty in shadow in NE during 2010–2018 (left panel) (detailed data in Supplementary Table S2) and CH_4_ emission increment relative to previous year (right panel).

The original Article has been corrected.

