# Supplementary material for: Atmospheric observations suggest methane emissions in north-eastern China growing with natural gas use
Source: Sci Rep. 2022 Nov 17;12:18587. doi: 10.1038/s41598-022-19462-4 (PMC9672054; doi:10.1038/s41598-022-19462-4)
Supplement: Supplementary file 1 — Supplementary Information. [file 41598_2022_19462_MOESM1_ESM.pdf]

Atmospheric Observations Suggest Methane Emissions in North-Eastern China Growing with Natural Gas Use

Fenjuan Wang[1,2], Shamil Maksyutov[1], Rajesh Janardanan[1], Aki Tsuruta[3], Akihiko Ito[1], Isamu Morino[1], Yukio Yoshida[1], Yasunori Tohjima[1], Johannes W. Kaiser[4], Xin Lan[5,6], Yong Zhang[7], Miao Liang[7], Ivan Mammarella[8], Jost V. Lavric[9] and Tsuneo Matsunaga[1]

[1] National Institute for Environmental Studies, Tsukuba, Japan

[2] National Climate Centre, China Meteorological Administration, Beijing, China

[3] Finnish Meteorological Institute, Helsinki, Finland

[4] Deutscher Wetterdienst, Offenbach, Germany

[5] Cooperative Institute for Research in Environmental Sciences, University of Colorado Boulder, Boulder, CO, USA

[6] National Oceanic and Atmospheric Administration, Global Monitoring Laboartory, Boulder, USA

[7] Meteorological Observation Center, China Meteorological Administration, Beijing, China

[8] University of Helsinki, Helsinki, Finland

[9] Max Planck Institute for Biogeochemistry, Jena, Germany

Supplementary Information

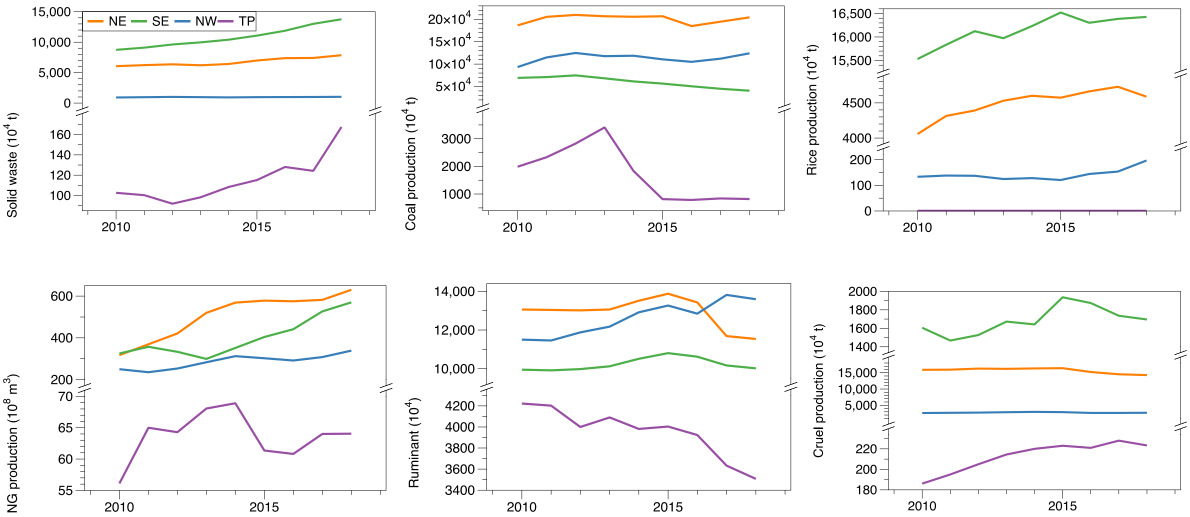

Fig. S1 The changes in the total activity data for major source sectors (coal, waste, rice, ruminant, NG and oil) of anthropogenic CH_4 emissions in the four regions of China during 2000–2018, data from China National Statistic Yearbook[1].

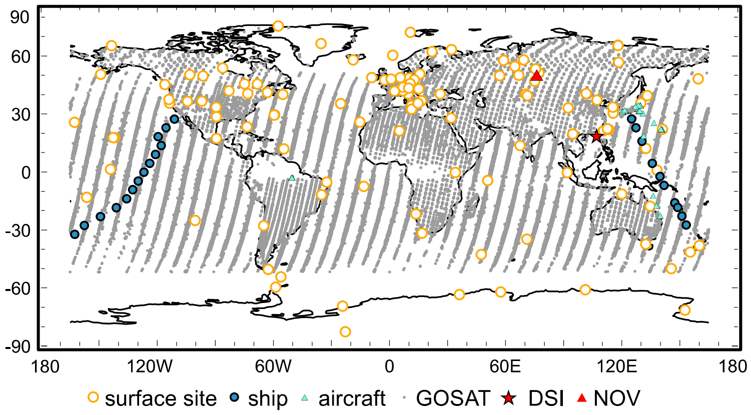

Fig. S2 GOSAT and WDCGG observation sites used in inversion. Ground-based atmospheric CH4 observation data are obtained from WDCGG[2], and aircraft and ships observations are from NIES. (Figure generated by ArcGIS Desktop 10.5.1, https://desktop.arcgis.com/en/arcmap/10.5/)

S3 Evaluation of inverse model by independent observation

Independent evaluation of the inverse model was made by observations at Dongsha Island (DSI) (20.7N, 116.7E) and Novosibirsk (NOV) (55N, 83E), location shown in Fig.S1. Bias and RMSE between measurement data and modelled concentrations with posterior fluxes decrease obviously in both sites, compared to modelled concentrations with prior fluxes (Supplementary material S1). DSI is a fixed monitoring station at Dongsha Island (Taiwan), and flask sampling was taken weekly from March 2010 to December 2017. Monthly flask sampling using research flights was taken above a pine forest near Novosibirsk in Russia (NOV) from February 2009 to December 2015 (more details of measurement are described by Sasakawa et al.[3]). The measurements from DSI and NOV are not included in the inversion. We conducted a simulation with the prior fluxes and posterior fluxes and compared the modelled concentrations with the observations at both sites as shown in Table 1. Correlations between measurement data, and modelled concentrations with inverse optimized fluxes increase at both sites.

Table S1 Bias, RMSE (root mean square error) and correlations between measurement data and modelled concentrations at NOV and DSI.

	NOV		DSI	
	prior	posterior	prior	posterior
Bias (ppb)	-28.67	1.44	-7.88	-4.75
RMSE (ppb)	28.06	24.29	32.23	27.68
Correlation	0.77	0.81	0.85	0.87

S4 The NG production, consumption volume, loss in urban areas, and pipelines length are collected from China statistic book and China city statistic book.

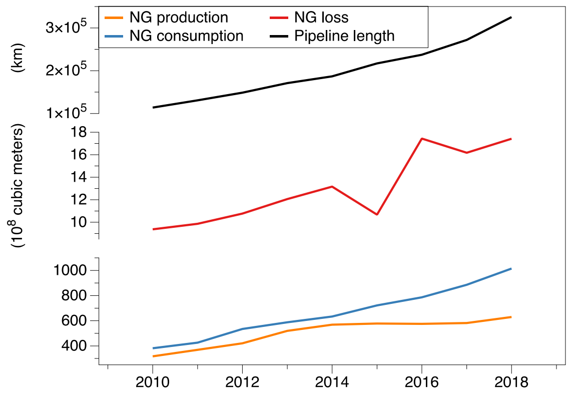

Fig. S3 Length of the pipeline, NG production, consumption, and loss in NE from 2010-2018.

Table S2 Estimated CH_4 emissions from NG (up and low range) and total CH_4 emission in NE during 2010-2018 in Fig.4.

Year	NG emissions (up range) (Tg CH_4 yr^{-1})	NG emissions (low range) (Tg CH_4 yr^{-1})	Total emissions (Tg CH_4 yr^{-1})
2010	1.13	1.82	20.73
2011	1.24	2.04	19.55
2012	1.39	2.32	20.53
2013	1.62	2.74	22.66
2014	1.76	2.99	23.58
2015	1.63	2.90	24.08
2016	2.10	3.39	26.57
2017	2.05	3.38	25.52
2018	2.23	3.69	26.91

Reference

1. *China City Statistical Yearbook*. (China Statistics Press, 2010-2018).
2. WDCGG CH4 data version 2019-09-19_11-36-27, WDCGG Global Network: https://gaw.kishou.go.jp, reference data: 2019-10-8
3. Sasakawa, M. *et al.* Continuous measurements of methane from a tower network over Siberia. *Tellus Series B-Chemical and Physical Meteorology* **62**, 403-416, doi:10.1111/j.1600-0889.2010.00494.x (2010).

S4 Observation data information

Table S2. List of observations used in this inversion, sites information and data file references.

GAW ID	Station/Mobile	Country /territory	Contributor	Plat-form	Sampling		Reference
	*** : Mobile**				**Type**	**Data set version**	**
ABP	Arembepe	Brazil	NOAA	surface	flask	0002-3003-1002-01-02-3001_2019-07-31-0735	6
AIA	Aircraft (over Bass Strait and Cape Grim)*	Australia	CSIRO	aircraft	flask	0016-8003-1002-05-02-9999_2019-07-19-1754	15
ALT	Alert	Canada	NOAA	surface	flask	0002-4001-1002-01-02-3001_2019-07-31-0735	6
ALT	Alert	Canada	ECCC	surface	insitu	0020-4001-1002-01-01-9999_2019-09-02-1446	6
AMS	Amsterdam Island	France	LSCE	surface	insitu	0045-1010-1002-01-01-9999_2018-09-17-1432	
AMT	Argyle (ME)	United States of America	NOAA	ship	flask	0002-4017-1002-01-02-3001_2019-07-31-0735	6
AMY	Anmyeon-do	Republic of Korea	KMA	surface	insitu	0039-2014-1002-01-01-9999_2018-09-07-1535	9
AOA	Aircraft (Western North Pacific)*	Japan	JMA	aircraft	flask	0001-8002-1002-05-02-9999_2019-09-03-1340	20
ARH	Arrival Heights	New Zealand	NIWA	surface	flask	0057-7009-1002-01-02-9999_2019-06-28-1226	13
ASC	Ascension Island	United Kingdom of Great Britain and Northern Ireland	NOAA	surface	flask	0002-1007-1002-01-02-3001_2019-07-31-0735	6
ASK	Assekrem	Algeria	NOAA	surface	flask	0002-1003-1002-01-02-3001_2019-07-31-0735	6
AZR	Serreta (Terceira)	Portugal	NOAA	surface	flask	0002-6047-1002-01-02-3001_2019-07-31-0735	6
BAL	Baltic Sea	Poland	NOAA	surface	flask	0002-6009-1002-01-02-3001_2019-07-31-0735	6
BHD	Baring Head	New Zealand	NOAA	surface	flask	0002-5012-1002-01-02-3001_2019-07-31-0735	6
BHD	Baring Head	New Zealand	NIWA	surface	insitu	0057-5012-1002-01-01-9999_2019-07-03-1027	
BKT	Bukit Kototabang	Indonesia	NOAA	surface	flask	0002-5007-1002-01-02-3001_2019-07-31-0735	6
BKT	Bukit Kototabang	Indonesia	BMKG	surface	insitu	0010-5007-1002-01-01-9999_2019-06-07-0922	11
BME	St. David's Head	United Kingdom of Great Britain and Northern Ireland	NOAA	surface	flask	0002-4029-1002-01-02-3001_2019-07-31-0735	6
BMW	Tudor Hill (Bermuda)	United Kingdom of Great Britain and Northern Ireland	NOAA	surface	flask	0002-4030-1002-01-02-3001_2019-07-31-0735	6
BRW	Barrow (AK)	United States of America	NOAA	surface	flask	0002-4003-1002-01-02-3001_2019-07-31-0735	6

BRW	Barrow (AK)	United States of America	NOAA	ship	insitu	0002-4003-1002-01-01-3001_2019-07-31-0930	7
BSC	Constanta (Black Sea)	Romania	NOAA	surface	flask	0002-6043-1002-01-02-3001_2019-07-31-0735	6
BSL	Pacific Ocean*	New Zealand	NIWA	surface	flask	0057-8023-1002-06-02-9999_2018-01-26-1037	13
CBA	Cold Bay (AK)	United States of America	NOAA	surface	flask	0002-4005-1002-01-02-3001_2019-07-31-0735	6
CFA	Cape Ferguson	Australia	CSIRO	surface	flask	0016-5010-1002-01-02-9999_2019-07-19-1754	18
CGO	Cape Grim	Australia	NOAA	surface	flask	0002-5011-1002-01-02-3001_2019-07-31-0735	6
CGR	Capo Granitola	Italy	ISAC	surface	insitu	0037-6048-1002-01-01-9999_2019-07-31-1323	5***
CHL	Churchill	Canada	ECCC	surface	flask	0020-4004-1002-01-02-9999_2019-09-02-1446	
CHL	Churchill	Canada	ECCC	surface	insitu	0020-4004-1002-01-01-9999_2019-09-02-1446	
CHM	Chibougamau	Canada	ECCC	surface	insitu	0020-4012-1002-01-01-9999_2019-09-02-1446	
CHR	Christmas Island	Kiribati	NOAA	surface	flask	0002-5006-1002-01-02-3001_2019-07-31-0735	6
CMN	Monte Cimone	Italy	IAFMS	surface	insitu	0029-6042-1002-01-01-9999_2019-04-29-1605	
CMN	Monte Cimone	Italy	UNIURB	surface	insitu	0074-6042-1002-01-01-9999_2018-04-19-0909	
COI	Cape Ochiishi	Japan	NIES	surface	insitu	0053-2008-1002-01-01-9999_2019-03-06-1319	19
CPA	Cholpon-Ata	Kyrgyzstan	Kyrgyzhydromet	surface	flask	0002-1009-1002-01-02-3001_2019-07-31-0735	6
CPT	Cape Point	South Africa	NOAA	surface	insitu	0066-1009-1002-01-01-9999_2019-03-13-1825	
CPT	Cape Point	South Africa	SAWS	surface	insitu	0066-1009-1002-01-01-9999_2019-03-13-1825	
CRI	Cape Rama	India	CSIRO	surface	flask	0016-2036-1002-01-02-9999_2019-07-19-1754	12
CRZ	Crozet	France	NOAA	surface	flask	0002-1011-1002-01-02-3001_2019-07-31-0735	6
CYA	Casey	Australia	CSIRO	ship	flask	0016-7004-1002-01-02-9999_2019-07-19-1754	4
DRP	Drake Passage*	United States of America	NOAA	surface	flask	0002-8044-1002-06-02-3001_2019-07-31-0735	6
ECO	Lecce Environmental-Climate Observatory	Italy	ISAC	surface	insitu	0037-6055-1002-01-01-9999_2019-01-28-2347	
EGB	Egbert	Canada	ECCC	aircraft	insitu	0020-4018-1002-01-01-9999_2019-09-02-1446	
EIC	Easter Island	Chile	NOAA	surface	flask	0002-3004-1002-01-02-3001_2019-07-31-0735	6
EOM	CONTRAIL*	Japan	NIES	surface	flask	0053-8007-1002-05-02-1501_2019-08-06-2047	14
ESP	Estevan Point	Canada	ECCC	surface	flask	0020-4013-1002-01-02-9999_2019-09-02-1446	
ESP	Estevan Point	Canada	ECCC	surface	insitu	0020-4013-1002-01-01-9999_2019-09-02-1446	
ETL	East Trout Lake	Canada	ECCC	surface	insitu	0020-4007-1002-01-01-9999_2019-09-02-1446	
FSD	Fraserdale	Canada	ECCC	tower	insitu	0020-4011-1002-01-01-9999_2019-09-02-1446	
GAT	Gartow	Germany	DWD	surface	insitu	0019-6058-1002-02-01-6342_2019-08-02-1548	

GMI	Guam (Mariana Island)	United States of America	NOAA	surface	flask	0002-5004-1002-01-02-3001_2019-07-31-0735	6
GPA	Gunn Point	Australia	CSIRO	surface	flask	0016-5008-1002-01-02-9999_2019-07-19-1754	18
GSN	Gosan	Republic of Korea	GERC	surface	insitu	0052-2025-1002-01-01-9999_2018-03-15-1245	
HAT	Hateruma Island	Japan	NIES	ship	insitu	0053-2031-1002-01-01-9999_2019-03-06-1319	19
HBA	Halley	United Kingdom of Great Britain and Northern Ireland	NOAA	surface	flask	0002-7008-1002-01-02-3001_2019-07-31-0735	6
HPB	Hohenpeissenber g	Germany	NOAA	tower	flask	0002-6028-1002-01-02-3001_2019-07-31-0735	6
HPB	Hohenpeissenber g	Germany	DWD	surface	insitu	0019-6028-1002-02-01-6132_2019-08-02-1548	
HUN	Hegyhatsal	Hungary	NOAA	aircraft	flask	0002-6034-1002-01-02-3001_2019-07-31-0735	6
ICE	Storhofdi	Iceland	NOAA	surface	flask	0002-6007-1002-01-02-3001_2019-07-31-0735	6
IZO	Izaña (Tenerife)	Spain	NOAA	surface	flask	0002-1002-1002-01-02-3001_2019-07-31-0735	6
IZO	Izaña (Tenerife)	Spain	AEMET	surface	insitu	0003-1002-1002-01-01-9999_2018-11-05-2322	10
JFJ	Jungfraujoch	Switzerland	Empa	surface	insitu	0023-6036-1002-01-01-9999_2019-05-17-2201	23
KEY	Key Biscane (FL)	United States of America	NOAA	surface	flask	0002-4033-1002-01-02-3001_2019-07-31-0735	6
KM W	Kollumerwaard	Netherlands	RIVM	surface	insitu	0063-6015-1002-01-01-9999_2018-07-09-1357	
KUM	Cape Kumukahi (HI)	United States of America	NOAA	surface	flask	0002-5003-1002-01-02-3001_2019-07-31-0735	6
KZD	Sary Taukum	Kazakhstan	NOAA	surface	flask	0002-2005-1002-01-02-3001_2019-07-31-0735	6
KZM	Plateau Assy	Kazakhstan	NOAA	surface	flask	0002-2007-1002-01-02-3001_2019-07-31-0735	6
LAU	Lauder	New Zealand	NIWA	surface	flask	0057-5014-1002-01-02-9999_2018-07-17-1223	13
LEF	Park Falls (WI)	United States of America	NOAA	tower	flask	0002-4015-1002-01-02-3001_2019-07-31-0735	6
LIN	Lindenberg	Germany	DWD	surface	insitu	0019-6057-1002-02-01-6099_2019-08-02-1548	
LLB	Lac La Biche (Alberta)	Canada	ECCC	surface	flask	0002-4006-1002-01-02-3001_2019-07-31-0735	6
LLB	Lac La Biche (Alberta)	Canada	NOAA	surface	insitu	0020-4006-1002-01-01-9999_2019-09-02-1446	
LLN	Lulin	Taiwan, Province of China	NOAA	surface	flask	0002-2032-1002-01-02-3001_2019-07-31-0735	6
LMP	Lampedusa	Italy	NOAA	surface	flask	0002-6051-1002-01-02-3001_2019-07-31-0735	6
LMP	Lampedusa	Italy	ENEA	surface	flask	0024-6051-1002-01-02-9999_2019-09-02-1411	3
LMT	Lamezia Terme	Italy	ISAC	surface	insitu	0037-6054-1002-01-01-9999_2019-03-07-2014	
MAA	Mawson	Australia	CSIRO	surface	flask	0016-7005-1002-01-02-9999_2019-07-19-1754	18
MEX	Mex High Altitude Global Climate	Mexico	NOAA	surface	flask	0002-4034-1002-01-02-3001_2019-07-31-0735	6

	Observation Center						
MHD	Mace Head	Ireland	NOAA	surface	flask	0002-6016-1002-01-02-3001_2019-07-31-0735	6
MHD	Mace Head	Ireland	LSCE	surface	insitu	0045-6016-1002-01-01-9999_2018-09-17-1432	
MID	Sand Island	United States of America	NOAA	surface	flask	0002-5001-1002-01-02-3001_2019-07-31-0735	6
MKN	Mt. Kenya	Kenya	NOAA	surface	flask	0002-1005-1002-01-02-3001_2019-07-31-0735	6
MLO	Mauna Loa (HI)	United States of America	NOAA	surface	insitu	0002-5002-1002-01-01-3001_2019-07-31-0930	7
MLO	Mauna Loa (HI)	United States of America	CSIRO	surface	flask	0016-5002-1002-01-02-9999_2019-07-19-1754	4
MNM	Minamitorishima	Japan	JMA	ship	insitu	0001-2029-1002-01-01-9999_2019-09-03-1340	21
MQA	Macquarie Island	Australia	CSIRO	surface	flask	0016-5015-1002-01-02-9999_2019-07-19-1754	18
NAT	Natal	Brazil	NOAA	surface	flask	0002-3001-1002-01-02-3001_2019-07-31-0735	6
NMB	Gobabeb	Namibia	NOAA	ship	flask	0002-1008-1002-01-02-3001_2019-07-31-0735	6
NWR	Niwot Ridge - T-van (CO)	United States of America	NOAA	aircraft	flask	0002-4023-1002-01-02-3001_2019-07-31-0735	6
ORL	Aircraft: Orleans*	France	LSCE	surface	flask	0045-8047-1002-05-02-9999_2018-09-17-1432	
OXK	Ochsenkopf	Germany	NOAA	surface	flask	0002-6022-1002-01-02-3001_2019-07-31-0735	6
PAL	Pallas	Finland	NOAA	ship	flask	0002-6004-1002-01-02-3001_2019-07-31-0735	6
PAL	Pallas	Finland	FMI	surface	insitu	0025-6004-1002-01-01-9999_2019-07-01-1911	16
PDI	Pha Din	Viet Nam	VNMHA	aircraft	insitu	0051-2035-1002-01-01-9999_2019-06-12-1820	8
PIP	Aircraft (off the Pacific coast of Sendai)*	Japan	TU	surface	flask	0070-8043-1002-05-02-9999_2019-07-22-1029	21
POC	Pacific Ocean*	United States of America	NOAA	surface	flask	0002-8034-1002-06-02-3001_2019-07-31-0735	6
PRS	Plateau Rosa	Italy	RSE	surface	insitu	0064-6039-1002-01-01-9999_2019-06-28-0828	
PSA	Palmer Station	United States of America	NOAA	surface	flask	0002-7003-1002-01-02-3001_2019-07-31-0735	6
PTA	Point Arena (CA)	United States of America	NOAA	surface	flask	0002-4025-1002-01-02-3001_2019-07-31-0735	6
PUY	Puy de Dôme	France	LSCE	tower	insitu	0045-6040-1002-01-01-9999_2018-09-17-1432	
RGL	Ridge Hill	United Kingdom of Great Britain and Northern Ireland	UNIVBRIS	surface	insitu	0077-6020-1002-02-01-6091_2019-07-11-1602	
RPB	Ragged Point	Barbados	NOAA	ship	flask	0002-4036-1002-01-02-3001_2019-07-31-0735	6
RYO	Ryori	Japan	JMA	ship	insitu	0001-2012-1002-01-01-9999_2019-09-03-1340	21
SAN	Santarem*	Brazil	INPE	surface	flask	0036-8004-1002-05-02-9999_2018-03-07-1431	

SDZ	Shangdianzi	China	NOAA	surface	flask	0002-2011-1002-01-02-3001_2019-07-31-0735	6
SEY	Mahé	Seychelles	NOAA	surface	flask	0002-1006-1002-01-02-3001_2019-07-31-0735	6
SGP	Southern Great Plains E13 (OK)	United States of America	NOAA	surface	flask	0002-4026-1002-01-02-3001_2019-07-31-0735	6
SHM	Shemya Island	United States of America	NOAA	surface	flask	0002-4009-1002-01-02-3001_2019-07-31-0735	6
SMO	Samoa (Cape Matatula)	United States of America	NOAA	surface	flask	0002-5009-1002-01-02-3001_2019-07-31-0735	6
SPO	South Pole	United States of America	NOAA	surface	flask	0002-7011-1002-01-02-3001_2019-07-31-0735	6
SPO	South Pole	United States of America	CSIRO	surface	flask	0016-7011-1002-01-02-9999_2019-07-19-1754	4
SSL	Schauinsland	Germany	UBAG	surface	insitu	0071-6027-1002-01-01-9999_2019-06-07-1841	1
STM	Ocean Station M	Norway	NOAA	surface	flask	0002-6006-1002-01-02-3001_2019-07-31-0735	6
SUM	Summit	Denmark	NOAA	surface	flask	0002-6002-1002-01-02-3001_2019-07-31-0735	6
SYO	Syowa	Japan	TU	tower	flask	0002-7006-1002-01-02-3001_2019-07-31-0735	6
TAC	Tacolneston Tall Tower	United Kingdom of Great Britain and Northern Ireland	NOAA	tower	flask	0002-6019-1002-01-02-3001_2019-07-31-0735	6
TAC	Tacolneston Tall Tower	United Kingdom of Great Britain and Northern Ireland	UNIVBRIS	aircraft	insitu	0077-6019-1002-02-01-6186_2019-07-11-1602	
TAP	Tae-ahn Peninsula	Republic of Korea	NOAA	surface	flask	0002-2013-1002-01-02-3001_2019-07-31-0735	6
TDA	Aircraft (over Japan and mainland)*	Japan	TU	surface	flask	0070-8042-1002-05-02-9999_2019-07-22-1029	21
TER	Teriberka	Russian Federation	MGO	surface	flask	0046-6003-1002-01-02-9999_2019-07-02-1719	
THD	Trinidad Head (CA)	United States of America	NOAA	surface	flask	0002-4022-1002-01-02-3001_2019-07-31-0735	6
TIK	Tiksi	Russian Federation	MGO	surface	flask	0002-2002-1002-01-02-3001_2019-07-31-0735	6
TIK	Tiksi	Russian Federation	NOAA	surface	insitu	0025-2002-1002-01-01-9999_2019-09-02-1407	
TIK	Tiksi	Russian Federation	FMI	surface	flask	0046-2002-1002-01-02-9999_2019-07-02-1719	
TLL	El Tololo	Chile	DMC	surface	insitu	0017-3005-1002-01-01-9999_2019-06-07-0922	17
USH	Ushuaia	Argentina	NOAA	surface	flask	0002-3007-1002-01-02-3001_2019-09-19-1114	6
UTA	Wendover (UT)	United States of America	NOAA	surface	flask	0002-4024-1002-01-02-3001_2019-07-31-0735	6
UUM	Ulaan Uul	Mongolia	NOAA	surface	flask	0002-2004-1002-01-02-3001_2019-07-31-0735	6
WIS	Sede Boker	Israel	NOAA	surface	flask	0002-6053-1002-01-02-3001_2019-07-31-0735	6

WKT	Moody (TX)	United States of America	NOAA	ship	flask	0002-4032-1002-01-02-3001_2019-07-31-0735	6
WLG	Mt. Waliguan	China	CMA	surface	flask	0002-2015-1002-01-02-3001_2019-07-31-0735	6
WPC	Western Pacific*	United States of America	NOAA	surface	flask	0002-8029-1002-06-02-3001_2019-07-31-0735	6
WSA	Sable Island	Canada	ECCC	surface	flask	0020-4019-1002-01-02-9999_2019-09-02-1446	
WSA	Sable Island	Canada	ECCC	surface	insitu	0020-4019-1002-01-01-9999_2019-09-02-1446	
YON	Yonagunijima	Japan	JMA	surface	insitu	0001-2028-1002-01-01-9999_2019-09-03-1340	21
ZEP	Zeppelin Mountain (Ny Ålesund)	Norway	NOAA	surface	flask	0002-6001-1002-01-02-3001_2019-07-31-0735	6
ZSF	Zugspitze-Schneefernerhaus	Germany	UBAG	surface	insitu	0071-6031-1002-01-01-9999_2019-06-29-2253	2
data direct from NIES (https://db.cger.nies.go.jp/portal/geds/atmosphericAndOceanicMonitoring)							
AZV	Azovo	Rassia	NIES	surface	insitu		24
BRZ	Berezorechka	Rassia	NIES	surface	insitu		24
DEM	Demyanskoe	Rassia	NIES	surface	insitu		24
IGR	Igrim	Rassia	NIES	surface	insitu		24
KSR	Karasevoe	Rassia	NIES	surface	insitu		24
NOY	Noyabrsk	Rassia	NIES	surface	insitu		24
SVV	Savvushka	Rassia	NIES	surface	insitu		24
VGN	Vaganovo	Rassia	NIES	surface	insitu		24
YAK	Yakutsk	Rassia	NIES	surface	insitu		24
COI	Cape Ochiishi	Japan	NIES	surface	insitu		19
HAT	Hateruma Island	Japan	NIES	surface	insitu		19

** list of refereces:

1. "Qualitatssicherungs-Handbuch des UBA-Messnetzes", UBA Texte 28/04, (Handbook for quality assurance for the measurement network for the Federal Environmental Agency, Germany), Dr.Klaus Nienerowski, Umweltbundesamt, Berlin, Juni 2004
2. own SOP for measurement of CO2, CH4 with Picarro CRDS, N2O, CO with Los Gatos QCL
3. Artuso, F., P. Chamard, S. Piacentino, A. di Sarra, D. Meloni, F. Monteleone, D. Sferlazzo, and F. Thiery, Atmospheric methane in the Mediterranean: analysis of measurements at the island of Lampedusa during 1995-2005, Atmos. Environ., 41, 3877-3888, 2007.
4. Bhattacharya, S.K., D.V. Borole, R.J. Francey, C.E. Allison, L.P. Steele, P. Krummel, R. Langenfelds, K.A. Masarie, Y.K. Tiwari and P.K. Patra, Trace gases and CO2 isotope records from Cabo de Rama, India, Current Science, 97, 9, 2009.
5. Cristofanelli P, Busetto M, Calzolari F, Ammoscato I, Gullì D, Dinoi A, et al.. Investigation of reactive gases and methane variability in the coastal boundary layer of the central Mediterranean basin. Elem Sci Anth. 2017;5:12. DOI: http://doi.org/10.1525/elementa.216
6. Dlugokencky, E.J., A.M. Crotwell, J.W. Mund, M.J. Crotwell, and K.W. Thoning (2019), Atmospheric Methane Dry Air Mole Fractions from the NOAA ESRL Carbon Cycle Cooperative Global Air Sampling Network, 1983-2018, Version: 2019-07 https://doi.org/10.15138/VNCZ-M766
7. Dlugokencky, E.J., A.M. Crotwell, P.M. Lang and J.W. Mund (2019), Atmospheric Methane Dry Air Mole Fractions from quasi-continuous measurements at Barrow, Alaska and Mauna Loa, Hawaii, 1986-2018, Version: 2019-03-04, Path: ftp://aftp.cmdl.noaa.gov/data/trace_gases/ch4/in-situ/surface/

8. For a short summary see Trinh Lan Phuong and Duong Hoang Long, Greenhouse gases measurements in Viet Nam, in Asia-Pacific GAW on Greenhouse Gases Newsletter vol 5, 33 - 38, 2014. available at http://www.wmo.int/pages/prog/arep/gaw/documents/The_5th_Asia_Pacifi_Newsletter_on_Greenhouse_Gases.pdf.
9. For methane the regional effects were briefed at http://www.wmo.int/pages/prog/arep/gaw/documents/Asian_GAW_GHGs_Newsletter_Vol4.pdf
10. Gomez-Pelaez, A.J., R. Ramos, V. Gomez-Trueba, R. Campo-Hernandez, E. Reyes-Sanchez: "GGMT-2015 Izaña station update: instrumental and processing software developments, scale updates, aircraft campaign, and plumbing design for CRDS" in GAW report (No. 229) of the "18th WMO/IAEA Meeting on Carbon Dioxide, Other Greenhouse Gases, and Related Measurement Techniques (GGMT) (La Jolla, CA, USA, 13-17 September, 2015)", edited by P. Tans and C. Zellweger, World Meteorological Organization, 125-131,2016
11. http://gaw.empa.ch/gawsis
12. Langenfelds, R.L., R.J. Francey, B.C. Pak, L.P. Steele, J. Lloyd, C.M. Trudinger and C.E. Allison, Interannual growth rate variations of atmospheric CO2 and its δ13C, H2, CH4 and CO between 1992 and 1999 linked to biomass burning, Glob. Biogeochem. Cycles, 16(3), 1048, doi:10.1029/2001GB001466, 2002.
13. Lowe at al., J. Geophys. Res., 96, 15455-15467, 1991
14. Machida T., H. Matsueda, Y. Sawa and Y. Niwa (2019), Atmospheric trace gas data from the CONTRAIL flask air sampling over the Pacific Ocean, Center for Global Environmental Research, NIES, DOI:10.17595/20190828.001.
15. Pak, B.C., Vertical structure of atmospheric trace gases over Southeast Australia, PhD Thesis, University of Melbourne, Australia, 273 pp. (available at the University of Melbourne ePrints Repository via http://www.lib.unimelb.edu.au/eprints/), 2000.
16. The GC system (CH4,CO) was audited in 2007, and Picarro based system for CO2, CH4 and CO in 2012. See GAWSIS (https://gawsis.meteoswiss.ch/)
17. The observations are supported by the Federal Office of Meteorology and Climatology MeteoSwiss through the project Capacity Building and Twinning for Climate Observing Systems (CATCOS) between the Swiss Agency for Development and Cooperation (SDC) and MeteoSwiss.
18. Thoning, K.W., P.P. Tans and W.D. Komhyr, Atmospheric carbon dioxide at Mauna Loa Observatory, 2, Analysis of the NOAA/GMCC data, 1974 - 1985, J. Geophys. Res., 94, 8549-8565, 1989.
19. Tohjima Y., Machida T., Utiyama M., Katsumoto M., Fujinuma Y., and Mksyutov S., 2002 : Analysis and persentation in situ atmospheric methane measurements from Cape Ochi-ishi and Hateruma Island. J. Geophys.107(D12),Pages ACH 8-1-ACH 8-11, https://doi.org/10.1029/2001JD001003
20. Tsuboi, K., et al., Evaluation of a new JMA aircraft flask sampling system and laboratory trace gas analysis system, Atmos. Meas. Tech., 6, 1257-1270, 2013.
21. Tsutsumi, Y., K. Mori, M. Ikegami, T. Tashiro, K. Tsuboi, (2006) Long-term trends of greenhouse gases in regional and background events observed during 1998-2004 at Yonagunijima located to the east of the Asian continent. Atmospheric Environment, 40, 5868-5879.
22. Umezawa, T., D. Goto, S. Aoki, K. Ishijima, P. K. Patra, S. Sugawara, S. Morimoto and T. Nakazawa, Variations of tropospheric methane over Japan during 1988-2010, Tellus B, 66, 23837, doi:10.3402/tellusb.v66.23837, 2014.
23. Zellweger C., L. Emmenegger, M. Firdaus, J. Hatakka, M. Heimann, E. Kozlova, T. G. Spain, M. Steinbacher, M. V. van der Schoot, B. Buchmann, 2016 - Assessment of recent advances in measurement techniques for atmospheric carbon dioxide and methane observations, Atmospheric Measurement Techniques, 9, 4737-4757, doi:10.5194/amt-9-4737-2016.
24. Sasakawa, M. et al. Continuous measurements of methane from a tower network over Siberia. Tellus Series B-Chemical and Physical Meteorology 62, 403-416, doi:10.1111/j.1600-0889.2010.00494.x (2010).

*** We acknowledge CNR-ISAC (www.isac.cnr.it) for making available the atmospheric methane data from CGR station (Italy) throughout GAW-WDCGG. CGR was built under the

I-AMICA Project funded by the Italian National Operation Program “ Ricerca e Competitività” (Research and Competitiveness) 2007–2013 (PON“R&C”). The WMO/GAW operations during the period 2015 - 2018 were supported by the Project of National Interest NEXTDATA funded by MIUR.
